# Effect of a Gluten-Free Diet on the Intestinal Microbiota of Women with Celiac Disease

**DOI:** 10.3390/antibiotics14080785

**Published:** 2025-08-02

**Authors:** M. Mar Morcillo Serrano, Paloma Reche-Sainz, Daniel González-Reguero, Marina Robas-Mora, Rocío de la Iglesia, Natalia Úbeda, Elena Alonso-Aperte, Javier Arranz-Herrero, Pedro A. Jiménez-Gómez

**Affiliations:** 1 Department of Pharmaceutical and Health Sciences, Universidad San Pablo CEU, Urbanización Montepríncipe, 28660 Boadilla del Monte, Spain; mm.morcillo@usp.ceu.es (M.M.M.S.); paloma.rechesainz@ceu.es (P.R.-S.); marina.robasmora@ceu.es (M.R.-M.); pedro.jimenezgomez@ceu.es (P.A.J.-G.); 2 Research Group “Food and Nutrition in Health Promotion (CEU-NutriFOOD)”, Departamento de Ciencias Farmacéuticas y de la Salud, Facultad de Farmacia, Universidad San Pablo CEU, Urbanización Montepríncipe, 28660 Boadilla del Monte, Spain; nubeda@ceu.es (N.Ú.); eaperte@ceu.es (E.A.-A.); 3 Departamento de Ciencias Médicas Básicas, Instituto de Medicina Molecular Aplicada (IMMA) Nemesio Díez, Medicine Faculty, Universidad San Pablo CEU, Urbanización Montepríncipe, 28660 Boadilla del Monte, Spain; j.arranz3@usp.ceu.es

**Keywords:** celiac disease, gluten-free diet, gut microbiota, cenoantibiogram, metagenomics, antibiotic resistance

## Abstract

**Background/Objectives**: Celiac disease (CD) is an autoimmune disorder characterized by small intestinal enteropathy triggered by gluten ingestion, often associated with gut dysbiosis. The most effective treatment is strict adherence to a gluten-free diet (GFD), which alleviates symptoms. This study uniquely integrates taxonomic, functional, and resistance profiling to evaluate the gut microbiota of women with CD on a GFD. **Methods**: To evaluate the long-term impact of a GFD, this study analyzed the gut microbiota of 10 women with CD on a GFD for over a year compared to 10 healthy controls with unrestricted diets. Taxonomic diversity (16S rRNA gene sequencing and the analysis of α and β-diversity), metabolic functionality (Biolog EcoPlates^®^), and antibiotic resistance profiles (Cenoantibiogram) were assessed. **Results**: Metagenomic analysis revealed no significant differences in taxonomic diversity but highlighted variations in the abundance of specific bacterial genera. Women with CD showed increased proportions of *Bacteroides, Streptococcus*, and *Clostridium*, associated with inflammation, but also elevated levels of beneficial genera such as *Roseburia*, *Oxalobacter*, and *Paraprevotella.* Despite no significant differences in metabolic diversity, higher minimum inhibitory concentrations (MICs) in women in the healthy control group suggest that dietary substrates in unrestricted diets may promote the proliferation of fast-growing bacteria capable of rapidly developing and disseminating antibiotic resistance mechanisms. **Conclusions**: These findings indicate that prolonged adherence to a GFD in CD supports remission of gut dysbiosis, enhances microbiota functionality, and may reduce the risk of antibiotic resistance, emphasizing the importance of dietary management in CD.

## 1. Introduction

Celiac disease (CD) is a systemic, immune-mediated, multifactorial disorder triggered by the ingestion of gluten and related prolamins in genetically predisposed individuals [1]. This disorder affects more than 7 million people in Europe and has a higher prevalence in women, with an estimated incidence of 17.4 cases per 100,000 women compared to 7.8 per 100,000 men [2]. CD is one of the few chronic diseases where the genetic component (*HLA-DQ2* and *HLA-DQ8*), the implicated autoantigen (tissue transglutaminase, tTG), and the environmental trigger (gluten) have been clearly identified [3].

Although CD has a strong hereditary component, with a familial recurrence rate of 10–15%, only 2–3% of individuals carrying the predisposition genes develop the disease, despite more than 30% of the global population carrying these genes and being exposed to gluten. This indicates that additional environmental factors are critical in its development [4]. These include a range of external factors that interact with the host’s immune system, microbiota, and genetic background. Studies such as TEDDY, EAT, and PRO-FILE have identified early-life viral infections (rotavirus), mode of delivery (cesarean section), early or delayed gluten introduction, and antibiotic use as potential triggers [5]. These factors may contribute to immune dysregulation by altering the gut microbiota composition, compromising intestinal barrier integrity, and facilitating abnormal immune responses to gluten peptides.

In the context of celiac disease, environmental triggers refer to non-genetic external factors that can initiate or exacerbate the autoimmune response in genetically predisposed individuals. These triggers include dietary elements, microbial exposures, pharmacological agents, and infections, especially during early life. Their cumulative effects may influence the timing of disease onset, the severity of intestinal damage, and the likelihood of extraintestinal complications.

Gluten, a storage protein found primarily in wheat, constitutes between 80% and 85% of the total protein in this cereal. Its most relevant fraction, gliadin, is resistant to complete digestion by gastric, pancreatic, and intestinal enzymes, generating large peptide fragments (up to 33 amino acids) in the small intestine that can trigger immune responses in genetically predisposed individuals [6]. Currently, the only effective therapeutic strategy for CD patients is strict adherence to a lifelong gluten-free diet (GFD) [7].

Numerous studies have shown that a GFD significantly improves the quality of life of celiac patients, reduces clinical symptoms, and prevents long-term complications. For example, Borghini et al. [8] reported improvements in nutrient absorption and a reduction in serum inflammatory markers in patients who strictly followed this diet. Similarly, Poslt Königová et al. [9] indicated that a GFD reduces the risk of autoimmune complications and malignancies associated with CD, while Aljada et al. [10] highlighted its role in preventing malnutrition, peripheral neuropathy, and other severe complications.

The gut microbiota, an ecosystem composed of approximately 100 trillion microorganisms, plays essential roles in human health, with alterations implicated in the development and progression of CD [11]. Key roles include the production of short-chain fatty acids (SCFAs), such as butyrate, propionate, and acetate, compounds generated by genera like *Prevotella*, which are crucial for maintaining a proper pH and possess anti-inflammatory properties [12,13]. In CD patients, dysbiosis may decrease SCFA production, contributing to inflammatory processes and exacerbating symptoms [13,14]. Additionally, genera like *Bifidobacterium* and *Lactobacillus* synthesize essential vitamins, whose deficiency is common in the dysbiosis associated with CD.

The microbiota also plays a role in protecting against pathogens by competing for nutrients and space, as well as generating bacteriocins with antimicrobial activity. Bacteria like *Akkermansia muciniphila* and *Lactobacillus* are essential in this function, which is affected in CD patients, increasing susceptibility to infections [15,16,17]. Moreover, genera like *Bacteroides* and *Enterococcus* participate in immune regulation, preventing excessive inflammation and autoimmune processes, while *Akkermansia muciniphila* contributes to mucus production, reinforcing the integrity of the intestinal barrier and limiting the entry of antigens and bacteria into the bloodstream [18,19,20]. In CD, these functions are compromised, exacerbating inflammation and intestinal damage.

Amplicon metagenomics has revolutionized the study of gut microbial communities, allowing detailed exploration of bacterial diversity in various conditions, such as CD. This approach provides precise information about the composition through the analysis of specific DNA regions, such as *16S rRNA.* This technique has proven essential for understanding how dietary and other factors can alter the gut microbiota [21].

On the other hand, the functional diversity of the gut microbiota is a key concept in microbial stability and is usually evaluated using tools like BIOLOG. Functional diversity implies that different bacterial species can carry out similar metabolic functions, ensuring the maintenance of intestinal health even in the face of alterations in bacterial composition. This is especially important in the context of diseases like CD, where dietary or environmental changes can affect the microbiota and its functionality.

Another relevant aspect is the study of microbial communities as biomarkers and potential reservoirs of resistance genes. Gut bacteria can harbor these genes and transfer them to other strains, thereby increasing the spread of resistance mechanisms. This information is crucial for predicting how clinical interventions, such as dietary changes or the use of antibiotics, can influence antimicrobial resistance [22].

Previous studies have described taxonomic changes in the gut microbiota of CD patients following a gluten-free diet (GFD) [11,13]. In this study, we use an integrative approach to assess microbial composition, metabolic function, and antibiotic resistance at the same time. Our goal is to evaluate how a long-term GFD affects the gut microbiota of women with CD compared to healthy women on a standard diet. We focus on taxonomic and functional diversity, as well as the microbiota’s response to clinically relevant antibiotics. This comprehensive analysis may improve understanding of the microbiota’s role in CD pathogenesis, immune regulation, and response to antimicrobial treatments.

## 2. Results

### 2.1. Study of the Microbial Community Functionality

**Figure 1:**
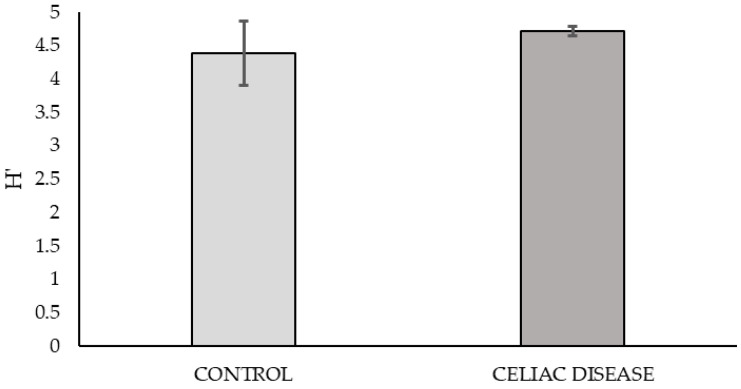
Metabolic diversity of bacterial communities from samples in the ‘Control vs. Celiac Disease’ study groups at 168 h post-inoculation (*p* = 0.361). Each bar represents Shannon’s Index (H′).

Based on the graph of nutrient consumption kinetics on the plate (Figure 1), the peak of metabolic activity was observed at 168 h post-bacterial inoculation. The metabolic diversity of the microbial community at this time, calculated using the mean Shannon Index from triplicate samples on the plate, showed no significant differences between the two study groups.

### 2.2. Response of the Bacterial Community to Specific Antibiotics

In the analysis of the antibiotic resistance profile of the bacterial community tested with the epsilon test (ε-test) under aerobic and anaerobic conditions, the MIC values obtained are detailed in Tables S1 and S2.

**Figure 2:**
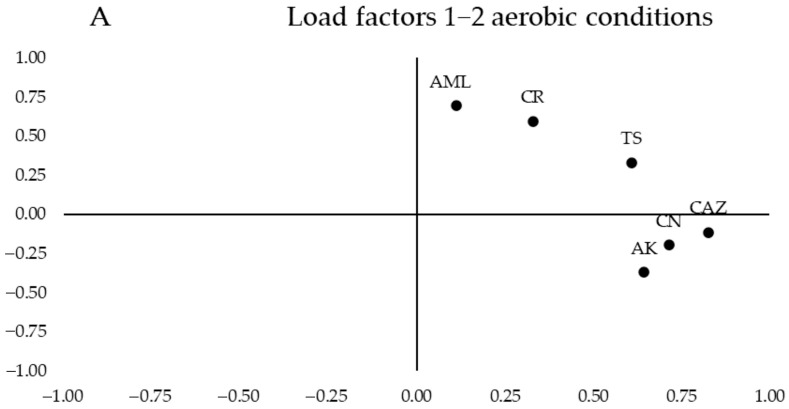

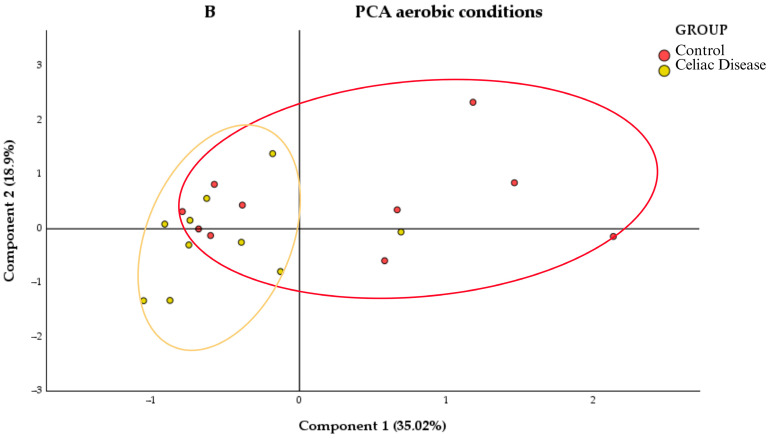
Principal component analysis (PCA) of the samples under aerobic conditions using different antibiotics: (**A**) 2D loading plot showing the influence of each antibiotic on the first two principal components, where each variable represents the antibiotics used under aerobic conditions: amikacin (AK), amoxicillin (AML), ceftazidime (CAZ), cefpirome (CR), gentamicin (CN), and sulfamethoxazole/trimethoprim (TS); (**B**) PCA plot representing the distribution and variation trends of the control group (Red) and the celiac group (Yellow) samples under aerobic conditions, in the 2D plane defined by the first two principal components, which explain the majority of the model (53.92% variance), in the context of the bacterial community.

In Figure 2B, a clear aggregation was observed on the left half of the values for the samples from women with CD on a gluten-free diet (red ellipse). In contrast, the control group of women with a normal diet clusters with a slight tendency of the values on the right half of the graph (yellow ellipse), although there is greater heterogeneity. Additionally, an area of overlap between the samples from both groups was visible.

**Figure 3:**
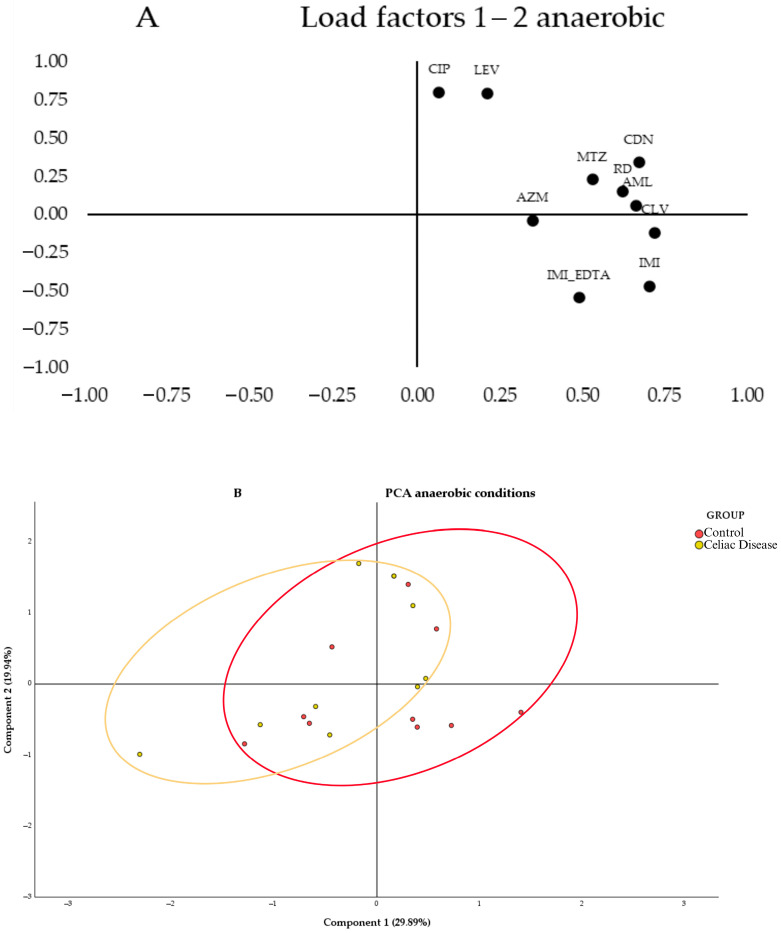
Principal component analysis (PCA) of the samples under anaerobic conditions using different antibiotics: (**A**) 2D loadings plot showing the influence of each antibiotic on the first two principal components, where each variable represents the antibiotics used under anaerobic conditions: ciprofloxacin (CIP), levofloxacin (LEV), clindamycin (CDN), rifampicin (RD), azithromycin (AZM), metronidazole (MTZ), imipenem (IMI), imipenem+EDTA (IMI_EDTA), and augmentin (AML_CLV); (**B**) PCA plot representing the distribution and variation trends of the control (Red) and celiac (Celiac) group samples in the space defined by the first two principal components, which explain the majority of the model (49.83% of the variance), within the context of the bacterial community.

In Figure 3A, the segregation of loadings was observed based on their nature and resistance mechanisms. Quinolones (CIP and LEV) are grouped in the upper left corner of the first quartile, while β-lactams (IMI, IMI_EDTA, and AML_CLV) are in the lower right corner of the same quartile. When testing antibiotics under anaerobic conditions (Figure 3B, no segregation of values between the study groups was observed, as both show greater heterogeneity in antibiotic susceptibility across their samples, with a large overlapping area. However, a slight trend was observed with the celiac group (red ellipse) leaning toward the left of the graph and the control group toward the right. Therefore, the control group samples tended to have higher MICs for the antibiotics CLV, IMI, CDN, and AML, as these samples were grouped in the positive region of Component 1, while the celiac group samples showed greater variability and lower MICs for these antibiotics, as they were grouped more toward the center and the negative region of Component 1.

These results were compared with a Student’s *t*-test to determine significant differences (*p*-value < 0.05) between the mean MICs of the two study groups. In antibiotic testing under aerobic conditions, significant differences in antibiotic resistance to CAZ and CR were observed, with the celiac group more sensitive to both antibiotics (Figure S2).

In antibiotic testing under anaerobic conditions, significant differences in MICs to IMI_EDTA were observed, with the celiac group more sensitive to this β-lactam (Figure S3).

Furthermore, in Tables S1 and S2 (Supplementary Material), we could observe the minimal inhibitory concentration (MIC) of different antibiotics for the bacteria present within cecal samples from the control and celiac groups under the given experimental conditions.

Resistance to ceftazidime was observed in both the control and celiac groups, with high MIC values (≥216 µg/mL). Examples include samples V9, V18, and V21 in the control group and V29 and V61 in the celiac group, all showing significant resistance. However, some samples exhibited sensitivity to ceftazidime, as indicated by lower MIC values (≤0.016 µg/mL), such as V42 and V58 in the control group and V60 and V73 in the celiac group. Overall, both groups demonstrated a similar pattern of elevated resistance to this antibiotic.

In contrast, resistance to cefpirome showed differences between the two groups. The control group exhibited higher MIC values overall (ranging from 0.064 to 256 µg/mL), with multiple samples demonstrating substantial resistance, such as V9, V18, and V21, each with MIC values of 256 µg/mL. On the other hand, the celiac group displayed a broader range of MIC values (0.016 to 12 µg/mL), suggesting lower resistance in general. Nonetheless, a few samples in the celiac group showed resistance, with one sample, V12, presenting a very high MIC value of 256 µg/mL, indicating variability within the group.

Resistance to ciprofloxacin was observed across both the control and celiac groups, with MIC values generally indicating a range of susceptibility. In the control group, MIC values ranged from 1 to 4 µg/mL, with most samples displaying low to moderate resistance. Similarly, in the celiac group, MIC values for ciprofloxacin ranged from 1 to 4 µg/mL, suggesting comparable resistance levels between the groups. Notably, a subset of samples in both groups exhibited lower MIC values, such as V42, V69, V21, V18, and V31 in the control group and V50, V64, V60, and V12 in the celiac group, with MIC values of 1 µg/mL, indicating potential sensitivity in certain cases.

Levofloxacin resistance followed a similar trend, with MIC values indicating moderate resistance in both groups. In the control group, MIC values ranged from 2 to 8 µg/mL, with samples such as V58, V78, and V21 exhibiting MIC values of 8 µg/mL. The celiac group showed a comparable range, with MIC values also falling between 2 and 8 µg/mL. For instance, samples V64, V82, V27, V73, and V29 in the celiac group had MIC values of 8 µg/mL, indicating resistance. Overall, resistance patterns to both ciprofloxacin and levofloxacin were similar in the control and celiac groups, with variability in susceptibility observed within each group.

Resistance to gentamicin was variable across the control and celiac groups. In the control group, MIC values ranged widely, from as low as 0.016 µg/mL (V58 and V31) to much higher values of 156 µg/mL in sample V42, indicating substantial resistance in some cases. The celiac group exhibited a similar range of MIC values, from highly susceptible samples such as V39, V50, V64, V27, and V29, each with MIC values of 0.016 µg/mL, to more resistant cases like V12 and V61, which had MIC values of 3 µg/mL. Overall, gentamicin resistance was observed in a subset of samples from both groups, but several samples demonstrated susceptibility, particularly in the celiac group.

For amikacin, resistance patterns were consistent with those observed for gentamicin, although higher MIC values were more frequently observed. In the control group, MIC values ranged from 0.016 µg/mL (V53) to 256 µg/mL in V18, indicating significant resistance. The celiac group also displayed a wide range of MIC values, with resistance evident in samples such as V12 and V29. However, lower MIC values were also observed in the celiac group, such as V39, V50, V82, and V60, with MIC values of 0.016 µg/mL, suggesting sensitivity in certain cases. Despite some variability, both groups exhibited a substantial proportion of samples with elevated MIC values, reflecting resistance to amikacin.

### 2.3. Metagenomics

After bacterial DNA sequencing, a total of 1,500,220 reads passed the quality control. The taxonomic analysis revealed 1,424,456 reads at the phylum level, 1,416,882 reads at the order level, 1,859,850 reads at the Family level, and 1,379,898 reads at the genus level.

#### 2.3.1. Alpha and Beta Diversity

**Figure 4:**
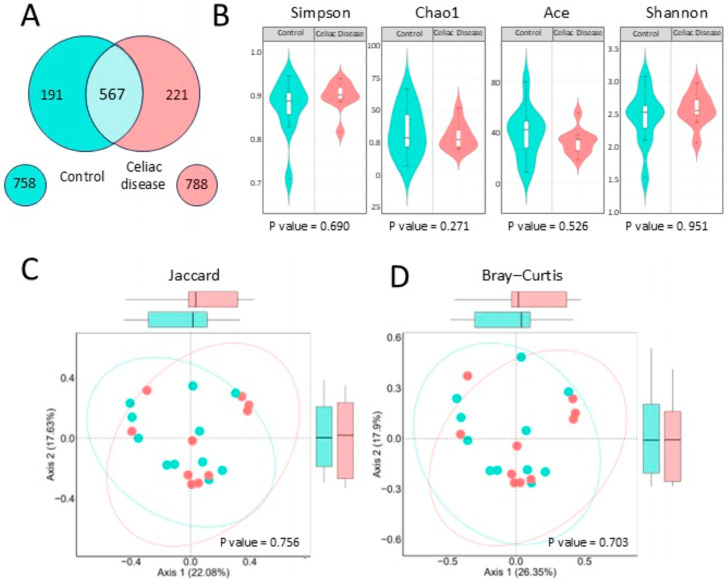
Bacterial diversity: (**A**) Venn diagram at the genus level. The light blue center shows the bacterial genera shared between both study groups. (**B**) Bacterial alpha diversity at the genus level, performed with the Simpson, ACE, Chao1, and Shannon tests. (**C**,**D**) Bacterial beta diversity at the genus level. On the left, the Jaccard dissimilarity index and, on the right, the Bray–Curtis index.

A preliminary comparison using a Venn diagram (Figure 4A) showed a similar number of bacterial genera between celiac patients (788) and controls (758), with 567 shared genera. Alpha diversity analyses (Figure 4B) revealed no significant differences between groups across multiple diversity indices, including Shannon (*p* = 0.951), Simpson (*p* = 0.690), Chao1 (*p* = 0.271), and ACE (*p* = 0.526).

The study of bacterial beta diversity (Figure 4C), using the Jaccard and Bray–Curtis’ index, shows that there are no significant differences in the composition and relative abundance of bacterial genera between the two groups. In the Bray–Curtis index analysis, there is a slight tendency of the celiac samples’ data to the right, in red, and the control samples to the left, in turquoise.

#### 2.3.2. Dominant Bacterial Phyla

**Figure 5:**
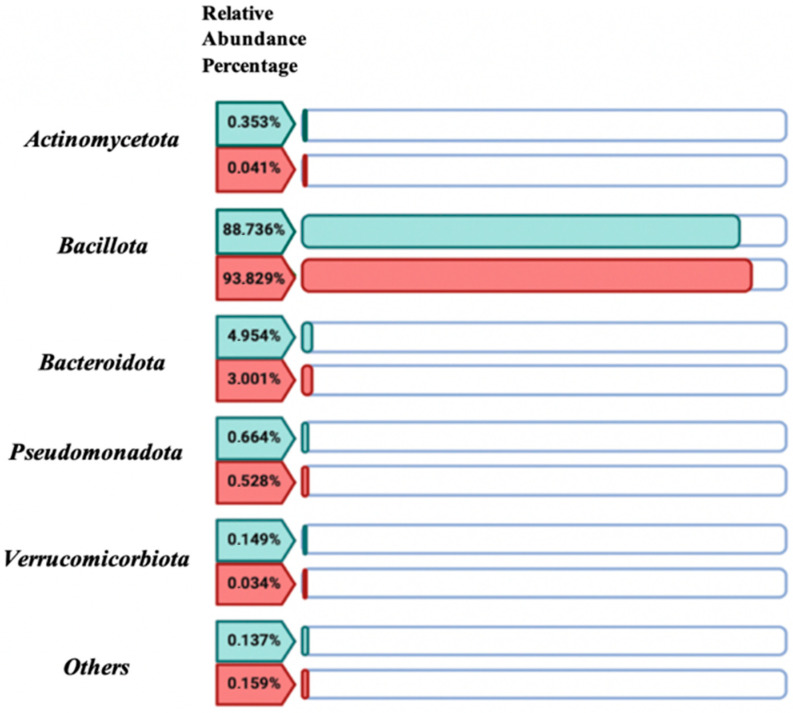
Relative abundance percentage of the 5 most representative phyla of the human gut microbiota in the control group (green) versus the women with celiac disease (red) study group. Image created with Biorender.

Through the comparative descriptive study of the relative abundance percentages of the five dominant bacterial phyla in the women with CD group versus the control group values (Figure 5), no significant differences (*p*-value > 0.05) were found between both study groups. The bacterial community was predominantly represented by the Bacillota phylum, accounting for 88.736% in the control group and 93.83% in the women with CD group.

#### 2.3.3. Biodiversity Index

Regarding the study of bacterial biodiversity through the Bacillota/Bacteroidetes and Bacteroides/*Prevotella* index (Table 1), in the case of the Bacillota/Bacteroidetes index, in the control group, the abundance of *Bacillota* is approximately 17.92 times higher than that of *Bacteroidetes,* considering that the data is in logarithmic scale (e.g., 10^1.253^ = 17.92). In contrast, in the women with CD study group, the abundance of *Bacillota* is approximately 31.21 times higher than *Bacteroidetes*.

Regarding the *Bacteroides*/*Prevotella index*, it is observed that, in the control group, the relative abundance of *Prevotella* is approximately 1.23 times higher than *Bacteroides*. In the women with CD study group, the abundance of *Bacteroides* is approximately 4.14 times higher than *Prevotella.*

#### 2.3.4. Dominant Bacterial Orders, Families, and Genera

**Figure 6:**
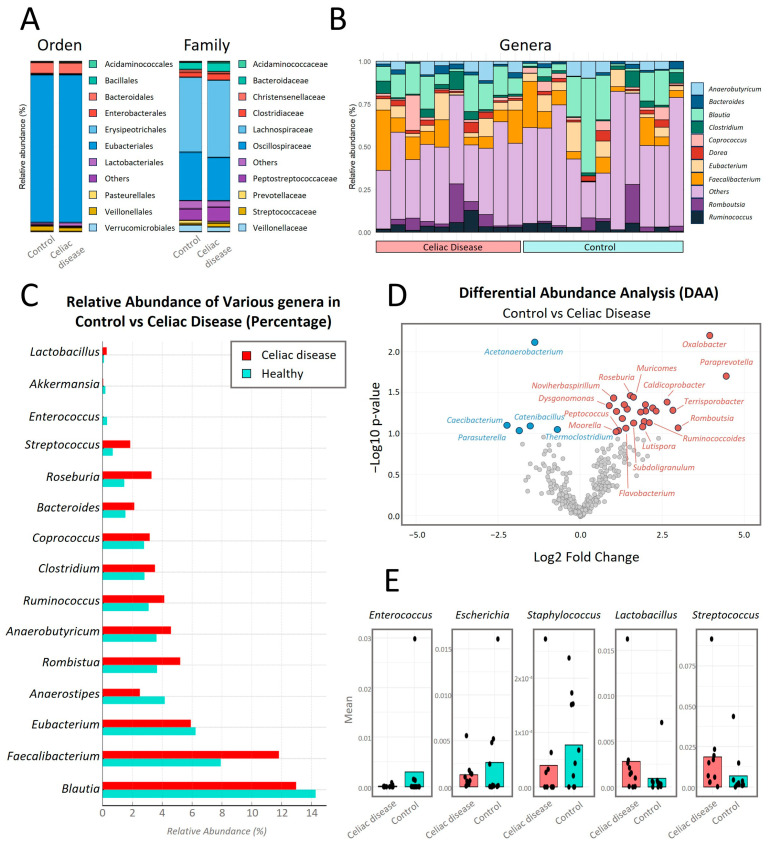
Comparative analysis of the microbiota composition at different taxonomic levels: (**A**) Comparative bar chart showing the top 10 families and orders with the highest relative abundance. (**B**) Stacked bar chart representing the relative abundance of the top 10 genera within each sample from the CD (red) and control groups (green). (**C**) Bar chart showing the top 10 genera with the highest relative abundance. (**D**) Volcano plot displaying the differential analysis of the relative abundance of bacterial genera using the LinDA method for the control group (in blue) and the celiac group (in red), with a cutoff *p*-value of 0.1. The *X*-axis represents the log2 fold change, indicating the magnitude of change in relative abundance. Positive values on this axis indicate greater abundance in the celiac group, while negative values indicate greater abundance in the control group. The *Y*-axis represents −log10 (*p*-value), where higher values indicate greater statistical significance. (**E**) Boxplot representing cultivable genera: *Enterococcus*, *Escherichia*, *Staphylococcus*, *Lactobacillus*, and *Streptococcus*.

In the comparative analysis of relative abundance at different taxonomic levels between both study groups (Figure 6), no significant statistical differences (*p* < 0.05) were observed at the taxonomic levels of Order and Family.

At the Order taxonomic level (Figure 6A), *Eubacteriales* is the bacterial Order with the highest relative abundance in both study groups, with a percentage around 88% in both groups. At the Family taxonomic level, the bacterial Family with the highest relative abundance in both groups is *Lachnospiraceae*, with a percentage of 44% in the control group versus 45% relative abundance in the women with CD group.

In the analysis of the top 10 genera with the highest intra-microbial relative abundance and comparison between both study groups (Figure 6B,C), the genus *Blautia* is the most abundant, with an average of 14% in the control group compared to 13% in the women with CD group. Secondly, the genus *Faecalibacterium* is increased in celiac patients with an average of 12% compared to 8% in the control group. *Roseburia* showed a significantly higher abundance (*p*-value = 0.035) in women with CD compared to the control group. *Bacteroides* showed a higher abundance in the celiac group, with a value of 2% compared to approximately 1.5% in the control group. *Coprococcus* was more abundant in the celiac group, with a value of 3% compared to approximately 2.5% in the control group. *Clostridium* also had a higher abundance in the celiac group, showing values of 3.5% compared to approximately 3% in the control group. *Ruminococcus* showed 4% relative abundance compared to approximately 3% in controls. *Anaerobutyricum* was slightly more abundant in celiacs compared to controls, with both groups having approximately 4%. *Romboutsia* showed values of 5%, compared to approximately 4% in controls. On the other hand, *Eubacterium* showed higher abundance in the control group compared to celiac patients.

In Figure 6D, significant differences in the relative abundance of certain bacterial genera between women with CD on a GFD (in red) and control women on a normal diet (in blue) were observed, using a *p*-value cutoff of 0.06. Genera such as *Oxalobacter*, *Paraprevotella*, *Roseburia*, *Muricomes*, and *Streptococcus* showed a significant increase in their relative abundance in women with CD compared to the control group with *p*-values of 0.0064, 0.0201, 0.0348, 0.0367, and 0.0558, respectively (Table S3). In contrast, genera like *Acetanaerobacterium* significantly decreased in women with CD compared to the control group, with a *p*-value of 0.0077 (Table S3).

In Figure 6E, the relative abundances of some of the cultivable genera (*Lactobacillus*, *Enterococcus*, *Escherichia*, *Staphylococcus*, and *Streptococcus)* are shown. The genera *Lactobacillus* and *Streptococcus* have higher abundance in celiac patients compared to controls, with the difference significant for *Streptococcus* (*p*-value = 0.056). Meanwhile, the genera *Enterococcus*, *Escherichia*, and *Staphylococcus* were more abundant in the control group.

## 3. Discussion

The impact of a GFD on gut microbiota was evaluated by studying 10 women diagnosed with CD who had been following a GFD for over a year, compared to a control group of 10 women on a normal diet. This comprehensive approach provided a deeper understanding of the microbial changes induced by the GFD, offering valuable insights into the interaction and functioning of gut bacteria. Although the GFD is essential for managing CD, it has been debated in several studies [10,23] due to its fiber deficiency, especially soluble fibers like beta-glucans (found in barley), and certain types of resistant starch, because of the exclusion of whole grains and some fiber-rich vegetables. In this context, there is evidence linking a reduced intake of certain types of dietary fiber required for bacterial fermentation and the production of SCFAs like butyrate and propionate to a reduced functional capacity of the microbiota. However, our results suggest that women with CD can maintain intestinal microbial functionality similar to women on a normal diet.

Similarly, the results related to functional diversity, particularly in substrate transformation capabilities, showed no significant differences between the two study groups. This suggests that the bacterial communities of both groups had the capacity to utilize a variety of substrates in a similar manner, indicating that dietary composition had a similar effect on individuals with different sensitivities to gluten when both groups had not been exposed to the allergen [24]. A recent study by Melini and Melini [25] showed that a GFD can be nutritionally adequate if it includes fiber-rich foods such as fruits, vegetables, legumes, and pseudocereals like quinoa or amaranth. These sources can compensate for the lack of fiber typically found in gluten-containing grains, supporting the idea that a well-planned GFD does not necessarily result in lower fiber intake and, therefore, a lower functionality of the microbiota [26].

The intestine may act as a reservoir for molecular mechanisms of antibiotic resistance. There is significant scientific interest in understanding the impact of diet on the preservation or control of the spread of resistance mechanisms shared by intestinal microbial communities. In this regard, the technique of the “cenoantibiogram”, defined as the analysis of the bacterial community’s response to antibiotics, does not aim to characterize each of the different mechanisms explaining the community’s response to antibiotics but rather to assess the overall phenotypic behavior of the intestinal microbial community. To precisely determine the resistance mechanisms involved, it would be essential to perform shotgun metagenomic sequencing.

In this context, the bacterial community of women with CD showed very homogeneous behavior both in aerobic conditions and in the absence of oxygen, with lower minimum inhibitory concentrations (MICs) to all tested antibiotics. These results suggested that prolonged adherence to a GFD in women with CD, compared to the control group, could influence the decrease in MICs to certain antibiotics. Conversely, in the control group’s bacterial community, there was a more heterogeneous antibiotic response in each sample, which suggests that a less restrictive diet may favor the proliferation of fast-growing bacteria, resulting in higher MICs.

Some studies [27,28] suggest that ultra-processed foods (UPFs), many of which are rich in gluten and not included in a GFD, contain simple carbohydrates. These simple sugars may favor the growth of fast-growing bacteria capable of proliferating rapidly due to the high availability of sugars. Fast-growing bacteria represent a significant public health challenge because of their ability to quickly develop and spread multiple antibiotic resistance mechanisms. Their high reproduction rates, horizontal gene transfer, biofilm formation, and rapid adaptation to antibiotic stress are key factors contributing to this problem [29]. Therefore, this observation could suggest that reducing the intake of these simple carbohydrates in GFDs could limit the growth of these bacteria, reducing MICs in the intestinal microbiota of celiac patients. When comparing metagenomic data, we observed a higher proportion of cultivable fast-growing bacteria, such as *Escherichia*, *Enterococcus*, and *Staphylococcus*, in the microbiota of the control women compared to the women with CD. These data support the hypothesis that adherence to a stricter GFD, compared to a normal diet, could lead to lower MICs for antibiotics and possibly lower proliferation of fast-growing bacteria.

In addition to the direct dietary effects of a GFD on the gut microbiota, previous studies have highlighted that the restoration of intestinal mucosal integrity and the reduction in intestinal permeability induced by GFD may also contribute to microbiota modulation. Notably, a significant proportion of patients with CD develop serum IgA and IgG antibodies against Saccharomyces cerevisiae, likely due to increased intestinal permeability. These antibodies tend to disappear following strict adherence to a GFD, as previously demonstrated. Interestingly, this immune feature has also been detected in asymptomatic individuals with potential or silent CD, suggesting that immune activation against microbial components may occur even in the absence of overt clinical symptoms. Therefore, it is plausible that GFD may influence gut microbiota composition not only through the exclusion of gluten and associated dietary changes but also by reducing immune stimulation secondary to improved intestinal barrier function. This dual mechanism may help create a more stable and less inflammatory microbial environment in individuals with CD.

In the control group, which consists of women on a normal, non-restrictive diet, there was a notable increase in resistance to third- and fourth-generation cephalosporins, as evidenced by elevated minimum inhibitory concentrations (MICs) to antibiotics such as ceftazidime and cefpirome. The intestinal microbiota can develop resistance to third- and fourth-generation cephalosporins through several mechanisms influenced by the complexity and diversity of the gut microbial community. One of the key factors is the production of β-lactamases by commensal bacteria. Extended-spectrum β-lactamases (ESBLs), produced by bacteria such as *Escherichia coli*, can hydrolyze advanced cephalosporins. These enzymes are often encoded by plasmid-borne genes, which can be horizontally transferred to other bacteria within the gut. Additionally, AmpC β-lactamases, produced by species such as *Enterobacter* and *Citrobacter,* also confer resistance to both third- and fourth-generation cephalosporins [30].

Resistance to ceftazidime is important for identifying beta-lactam resistance mechanisms. Both AmpC beta-lactamase hyperproduction and extended-spectrum beta-lactamases (ESBLs) lead to resistance to this cephalosporin. In this study, ceftazidime resistance was prioritized to infer the underlying resistance mechanisms. This information helps clarify the specific mechanism of driving beta-lactam resistance in bacterial populations, making ceftazidime resistance a critical focus in resistance studies [31].

In contrast, the group with CD following a gluten-free diet did not exhibit the same level of resistance, indicating that dietary factors might influence the gut microbiota. Horizontal gene transfer (HGT) is crucial for the spread of cephalosporin resistance within the gut microbiota. The gut acts as a reservoir for antibiotic resistance genes (ARGs), which can be transferred between commensal bacteria and potential pathogens through mechanisms such as conjugation, transformation, or transduction. This transfer process helps spread resistance traits throughout the microbial community. In addition to β-lactamase production and gene transfer, adaptive mechanisms like efflux pump overexpression and porin loss contribute to resistance. Bacteria such as *Pseudomonas* and *Klebsiella* may overexpress efflux pumps or modify their porins, leading to reduced drug uptake and increased resistance to cephalosporins [32]. While these mechanisms are more commonly found in pathogenic bacteria, they can also emerge in commensals under selective pressure.

Quinolone resistance in bacteria primarily arises through several mechanisms, including mutations in the genes encoding the target enzymes DNA gyrase (gyrA) and topoisomerase IV (parC). These enzymes are crucial for DNA replication, and mutations in their coding regions alter their structure, reducing quinolone binding affinity and making the drug less effective.

In addition, efflux pumps also contribute to resistance by actively pumping quinolones out of bacterial cells, reducing their intracellular concentration. The overexpression of efflux pumps like acrAB in *Enterobacteriaceae* can significantly enhance resistance. Another mechanism is the reduction in membrane permeability, where changes in the bacterial cell membrane limit the entry of quinolones into the cell, decreasing their effectiveness. Many intestinal bacteria, including *Pseudomonas aeruginosa* and *Enterobacteriaceae*, can overexpress efflux pumps that actively pump quinolones out of the bacterial cell [33].

Among the main acquired resistance mechanisms, plasmid-mediated mechanisms of resistance to quinolones (PMQRs) are particularly noteworthy. These mechanisms involve cytoplasmic proteins known as qnr (quinolone resistance proteins), which provide protection to topoisomerases (DNA gyrase and topoisomerase IV) by hindering the binding of quinolones. Additionally, the acetyltransferase enzyme aac (6’)-Ib-cr plays a role in inactivating quinolones through acetylation.

In a study, the group of women with CD exhibited higher minimum inhibitory concentrations (MICs) for fluoroquinolones compared to the control group, indicating a stronger resistance in the CD group. This suggests that changes in the gut microbiota, potentially influenced by dietary factors, may promote the development of quinolone resistance through these various resistance mechanisms. Several types of qnr have been described, including qnrA, qnrS, qnrB, qnrC, qnrD, qnrVC, and qnrE, each with its own allelic variants [34,35]. These proteins are often associated with extended-spectrum beta-lactamases (ESBLs), AmpC beta-lactamases, and carbapenemases, and they are typically carried on the same conjugative plasmids that confer multi-drug resistance. This facilitates the spread of resistance traits among bacterial populations, contributing to the broader challenge of antimicrobial resistance [36].

The two primary mechanisms of aminoglycoside resistance in *Enterobacterales* are enzymatic modification of the drugs or alteration of their target sites. Additionally, there is evidence of disruptions in bacterial penetration or active efflux mediated by pumps such as AcrD.

Aminoglycoside-modifying enzymes are classified into three major families: nucleotidyl (adenyl)-transferases, phospho-transferases, and acetyltransferases. Each family contains numerous variants that transfer AMP, phosphate, or acetyl-coenzyme A to specific positions on the aminoglycoside molecules, thereby inactivating their antibacterial activity. Aminoglycoside-modifying enzymes exhibit a specific and limited spectrum of activity. This means that they usually only confer resistance to certain aminoglycosides and do not cause cross-resistance (resistance to multiple different types of drugs in the same class) in the way beta-lactamases might [37]. The control group showed higher MICs for gentamicin and amikacin. The presence of high MICs for both gentamicin and amikacin suggests either the simultaneous action of multiple aminoglycoside-modifying enzymes or the contribution of target site mutations and efflux mechanisms. Further molecular characterization would be required to determine the exact resistance determinants.

Based on our results, a molecular investigation of the resistance mechanisms would be valuable, as elucidating these mechanisms is essential for understanding their substantial clinical implications. The intestinal microbiota acts as a reservoir for antibiotic resistance genes, which can transfer to pathogenic bacteria during infection, potentially leading to more severe clinical outcomes. Furthermore, the overgrowth of resistant bacteria can disrupt gut homeostasis, causing dysbiosis and increasing susceptibility to infections.

However, inherent difficulties in studying the intestinal microbiota and challenges in obtaining consistent results necessitate further studies to clarify these preliminary findings. Furthermore, standardizing protocols for bacterial DNA extraction from fecal samples and the subsequent bioinformatics processing of the obtained data can facilitate better interpretation of results across research studies [38,39]. There is a high scientific consensus stating that Gram-positive bacteria present more difficulty in lysis and proper DNA extraction [32]. In our results, high-quality sequencing and significant relative abundance of the Gram-positive phylum Bacillota were observed in both patient groups, indicating the suitability of the process used for bacterial DNA extraction from fecal samples [40].

A key finding was the result obtained for both alpha and beta diversity indices. Women with CD on a GFD did not show significant differences compared to the control group in either the composition or the bacterial richness at the genus level. This supports recent studies [7,41] that have demonstrated that a GFD can restore microbial balance in the intestines of celiac patients, whose intestinal bacterial dysbiosis is well-documented, thereby improving the gastrointestinal clinical manifestations of the disease. Similarly, other studies suggest that a GFD can restore alpha diversity, while beta diversity differences compared to individuals on a normal diet may persist. This would indicate that celiac patients on a GFD may have a similar amount or richness of bacterial species in their intestines as control individuals, while the exact species present may differ between the two study groups [42]. Our study did not observe these described differences, which is a positive indicator of the importance of a GFD in modulating the gut microbiota and its potential to improve the health of people with CD.

Biodiversity indices revealed a higher relative proportion of bacteria from the *Bacteroides* genus compared to *Prevotella* in women with CD. This increase in *Bacteroides* is documented in inflammatory gastrointestinal pathologies such as CD, Crohn’s disease, and ulcerative colitis [43]. A higher relative abundance of *Bacteroides* was also observed in the metagenomic study in the women with CD compared to the control group. This genus is described as pro-inflammatory, and an abundance of it may exacerbate gastrointestinal inflammatory conditions [44]. Lipopolysaccharides (LPSs) are components of the outer membrane of Gram-negative bacteria like *Bacteroides*. While high doses of LPS are known to induce a strong inflammatory response, recent research [45,46] has shown that exposure to small amounts of LPS may not trigger such acute inflammation as high doses, but sustained activation of TLR4 and NF-κB may maintain chronic production of pro-inflammatory cytokines like TNF-α, IL-1β, and IL-6.

In the case of the metagenomic results obtained for *Streptococcus*, another pro-inflammatory genus, a significantly higher abundance was observed in the women with CD compared to the control group. The study by Maciel-Fiuza et al. [47] highlights that *Streptococcus*, in combination with *Veillonella*, a genus also increased in the women with CD (Table S3), can exacerbate the production of pro-inflammatory cytokines like TNF-α, IL-8, IL-6, and IL-10, contributing to an inflammatory state in the intestines. It is important to note that, although general taxonomic diversity may be similar between women with CD and control women, alterations in specific bacterial genera may influence the inflammation and intestinal permeability characteristic of CD. These alterations may not manifest in daily symptoms if patients adhere to a GFD but highlight the importance of monitoring and maintaining a healthy balance in the gut microbiota to prevent long-term complications. Studies such as Spatola [48] have pointed out that not all celiac patients respond completely to a GFD, with a minority of patients (approximately 10–19%) suffering from non-responsive CD (NRCD), where the intestinal mucosa does not fully recover even after a year on a GFD, with persistent inflammation.

Therefore, the metagenomic results obtained in this study suggest that a GFD could restore bacterial balance in celiac patients, increasing the relative abundance of beneficial SCFA-producing bacteria, such as *Paraprevotella*, *Roseburia*, *Lactobacillus*, or *Faecalibacterium*, thus supporting other studies [10,49]. This could counteract the possible discussed fiber deficiency in a GFD in celiac patients, as evidenced by a similar substrate degradation capacity in celiac patients compared to the control group.

The increase in the relative abundance of the cultivable *Escherichia* genus and the significant rise in MICs for the tested antibiotic in anaerobiosis (IMI_EDTA) observed in the control group compared to the celiac group suggests that a non-restrictive diet increases the relative abundance of a bacterial genus known for its ability to acquire and spread antibiotic resistance genes, including carbapenemases, enzymes that degrade carbapenems. Dietary restrictions may limit exposure to certain dietary and environmental factors that favor the selection and proliferation of these resistant bacteria.

It is relevant to mention that the functional redundancy of the intestinal microbiota, a concept explaining that different bacterial species can perform similar metabolic functions, ensuring functional stability despite changes in bacterial composition [50], could explain why, despite significant differences in the relative abundance of specific bacterial genera, the overall functionality of the microbiota in women with CD is not compromised.

This study has some limitations that should be acknowledged. First, the sample size was relatively small, determined by the available resources and the complexity of the multi-omics approach. Nevertheless, the consistency of the results supports the validity of the findings and provides a solid foundation for future studies with larger cohorts. Second, although the original study design aimed to include both sexes, the decision to focus on women was based on practical considerations and the higher prevalence of CD in women (female-to-male ratio of approximately 1.5:1). This population represents a clinically relevant group with a greater disease burden, making it a strategic choice for initial investigation. Third, while including a group of patients with CD not adhering to a gluten-free diet could have improved the comparison, it was not considered ethically acceptable, as continued gluten exposure is known to cause mucosal damage and long-term health risks. Therefore, our comparison focused on patients in remission versus healthy controls to evaluate the long-term impact of the GFD.

## 4. Materials and Methods

### 4.1. Sample Collection and Inclusion Criteria

In 2022, a double-blind comparative study was conducted to analyze the gut microbiota of 10 women with diagnosed CD who were following a gluten-free diet for more than one year, compared to 10 control women following a normal diet. All participants were aged between 19 and 59 years. The study population was limited to adult women due to logistical constraints and to ensure feasibility within the available resources.

The samples were collected by the “Food and Nutrition in Health Promotion Research Group” (CEU-NutriFOOD, ref. C08/0720) at CEU San Pablo University (Madrid, Spain) as part of the cross-sectional study on women, VENCELIAQ-FOL. Women with CD were identified through the Spanish Celiac Disease and Gluten Sensitivity Association in Madrid (ACSG) by distributing an informational brochure via email, social media, and the association’s publications. For the recruitment of controls, the research team collaborated with volunteers who expressed interest in participating by learning about this study through CEU-NutriFOOD staff at CEU San Pablo University (Alcorcón, Madrid, Spain) and social media.

For the celiac group, inclusion criteria required the following: (1) a confirmed medical diagnosis of CD by a healthcare professional, (2) adherence to a gluten-free diet for more than one year, (3) absence of associated diseases, and (4) no use of nutritional supplements. For the control group, inclusion criteria included the following: (1) no diagnosis of any chronic disease, (2) no regular symptoms or signs of digestive disorders, and (3) no use of nutritional supplements.

To ensure the absence of confounding factors, exclusion criteria for both groups included the following: the presence of other digestive or autoimmune diseases (e.g., Crohn’s disease, irritable bowel syndrome, ulcerative colitis, colon cancer), and the use of immunosuppressive therapies, anti-inflammatory drugs, metformin, proton pump inhibitors, or antibiotics within the previous six months.

This information was collected through a structured screening questionnaire prior to inclusion to verify eligibility and confirmed during a 24 h dietary recall conducted by a trained dietitian.

Volunteers who did not meet the inclusion criteria were excluded, as well as women who were pregnant or breastfeeding. Additionally, for the control group, volunteers who tested positive for anti-tissue transglutaminase antibodies (AAtTGs) were excluded, as they might have undiagnosed CD.

A stool sample of the size of a walnut was collected in a sterile container, ensuring it was free from urine or water contamination. The container was sealed tightly and labeled appropriately. Participants were instructed to immediately wrap the container in aluminum foil and freeze the sample at home after collecting it. Regarding transport, the frozen sample was placed in an insulated bag with cold packs and delivered to the laboratory, where it was immediately stored at −80 °C until analysis.

### 4.2. Study of Microbial Community Functionality

A 1:9 dilution of each fecal sample was prepared in saline solution (0.45% NaCl) from 2 g of pure sample. It was homogenized with an Omni-Mixer homogenizer at 16,000 r.p.m. for two minutes. It was then centrifuged at 690× *g* for five minutes with a Hettich Zentrifugen centrifuge model Mikro 22R (Hettich GmbH, Tuttlingen, Germany), and the optical density was adjusted to 0.5 McFarland (>10^8^ CFU/mL of viable microorganisms) using a Densimat^®^ (bioMérieux, Marcy-l’Étoile, France). The Biolog Eco^®^ plates (Biolog Inc., Hayward, CA, USA) were loaded with 135 μL of supernatant per well. The plates were incubated for 168 h at 37 °C, with absorbance measurements taken every 24 h at 590 nm using the Asys UVM340 plate reader (Biochrom Ltd., Cambridge, UK) and the Micro WinTM V3.5 software (Mikrotek Laborsysteme GmbH, Overath, Germany).

### 4.3. Cenoantibiogram

From the same bacterial suspension obtained under the conditions described in the previous section, flooding inoculation was performed on Mueller–Hinton agar (Condalab^®^, Madrid, Spain), and the minimum inhibitory concentration (MIC) was evaluated in triplicate using antibiotic strips (ε-test). Under aerobic conditions, the following antibiotics were tested: amikacin (AK), amoxicillin (AML), ceftazidime (CAZ), cefpirome (CR), gentamicin (CN), and sulfamethoxazole/trimethoprim (TS) (BioMérieux^®^, Marcy l’Etoile, France). The same procedure was performed under anaerobic conditions for the following antibiotics: ciprofloxacin (CIP), levofloxacin (LEV), clindamycin (CDN), rifampicin (RD), azithromycin (AZM), metronidazole (MTZ), imipenem (IMI), imipenem + EDTA (IMI_EDTA), and augmentin (AML_CLV) (BioMérieux^®^, Marcy l’Etoile, France). The plates were incubated according to the manufacturer’s instructions. For MIC quantification, the most restrictive halo was used as the reference.

### 4.4. Statistical Analysis

A principal component analysis (PCA) was conducted, starting with a 2D projection of the factor loadings. The distribution of individuals in the PCA corresponds to the distribution of the factor loadings. To contrast this data, a Student’s *t*-Test was performed to assess significant differences in MIC values between the two study groups. All statistical analyses were performed using SPSS software (Version 30.0, IBM Corp, Armonk, NY, USA).

To assess significant differences in MIC values between the celiac and control groups, a Student’s t-Test was performed. All statistical analyses were conducted using SPSS software (Version 27.0, IBM Corp, Armonk, NY, USA).

The raw results obtained from the absorbance measurements of the Biolog Eco^®^ were corrected by subtracting the blank (corrected absorbance value). The r of AWCD (Average Well Color Development) was plotted against incubation time to obtain the growth curves of the microbial community in the wells of the plate. The point of incubation where microbial growth began to enter the stationary phase was selected for subsequent multivariate analyses. Additionally, using the corrected absorbance values at the selected incubation point as AWCD, the metabolic diversity of each sample was calculated using the following formula: $H\left(m\right)=-\sum q_{i}log2q_{i};$ q_i_ = n/N, where *n* is the corrected absorbance (AWCD) of each well, and *N* is the total absorbance of all wells.

### 4.5. Metagenomic Analysis

#### 4.5.1. Bacterial DNA Extraction

Microbial DNA was extracted using the REAL Microbiome Fecal DNA kit (Durviz, Valencia, Spain), and the protocol was optimized to improve the extraction quality. DNA purity and concentration were determined using the NanoDrop One Microvolume UV-Vis spectrophotometer (Thermo Fisher Scientific, Waltham, MA, USA).

#### 4.5.2. Amplification and Library Preparation

DNA amplification was performed using the 16S Barcoding Kit (SQK-RAB204, Oxford Nanopore Technology, Oxford, UK) following Matsuo et al. [51]. The primers used for amplifying the full 16S gene region (V1-V9) were the universal primers 27F (5′-AGAGTTTGATCMTGGCTCAG-3′) and 1492R (5′-TACGGYTACCTTGTTACGACTT-3′), with a unique sample barcode sequence attached. PCR was carried out in a thermal cycler with the following conditions: initial denaturation (1 min, 95 °C), 25 cycles of denaturation (20 s, 95 °C), annealing (30 s, 55 °C), and elongation (7 min, 65 °C).

The PCR product was purified using AMPure XP beads (Beckman Coulter, Indianapolis, IN, USA), and the library was prepared according to the 16S Barcoding Kit instructions.

#### 4.5.3. Sequencing

In total, 200 ng of a pooled library was loaded onto an R9.4.1 flow cell, and sequencing was carried out on the MinION Mk1B device (Oxford Nanopore Technologies, Oxford, UK) for 24 h using the MinKNOW analysis software (v23.11.4) with default settings. Demultiplexing, or obtaining signals by sample, was performed using the same software. The FAST5 sequencing data (voltage change signals) were automatically processed with the Dorado software (v7.2.13) embedded in the MinKNOW interface with default settings to obtain reads in FASTQ format. Additionally, the quality of the obtained reads was assessed, with a minimum Quality Score of 8.

#### 4.5.4. Processing and Analysis of Metagenomic Data

All the reads that passed quality control were isolated and used as input for the Fastq16S v2022.01.07 workflow from EPI2ME (Oxford Nanopore Technologies, Oxford, UK). This protocol uses the NCBI database to return an identification based on sequences analyzed with BLAST (https://blast.ncbi.nlm.nih.gov/Blast.cgi). Subsequently, normalization was performed using Total Sum Scaling (TSS) on the CSV reports exported by EPI2ME (v5.2.2), utilizing the R program language and the RStudio interface Version 2023.09.1+494 (RStudio Team, Vienna, Austria, 2023).

The TSS normalization was calculated using the following formula:

$$p_{ij}=\frac{x_{ij}}{\sum_{j\;=1}^{n}x_{ij}}\times 1,000,000$$

*p_ij_* is the normalized relative abundance of the genus *j* in the sample, *i*, *x_ij_* is the number of reads of the genus *j* in the sample *i*, and $\sum_{j=1}^{n}x_{ij}$ is the sum of reads in the sample *i*.

The alpha diversity indices at the taxonomic genus level, including the Shannon Indices ($H\;=\;-\;\sum_{i\;=\;1}^{s}p_{i}\ln{(p}_{i})$), Simpson ($D=\;1\;-\;\sum_{i\;=\;1}^{s}p_{i}^{2}$), Chao1 ($S_{est}=S_{obs}\;+\frac{F_{1}^{2}}{2F_{2}}$), and ACE ($S_{ACE}=S_{abund}+\frac{F_{1}}{C_{ACE}}$) were calculated in RStudio using the “MicrobiomeStat” analysis package to evaluate richness and diversity within the samples (intrasample). Additionally, beta diversity at the taxonomic genus level was calculated using Bray–Curtis dissimilarity ($BC_{ij}=\;1\;-\;\frac{2C_{ij}}{S_{i}\;+{\;S}_{j}}$) and Jaccard index ($J\;=\;\frac{A}{A\;+\;B\;+\;C}$), also calculated with “MicrobiomeStat” to compare diversity between different samples and study groups (intersample). To identify differences in taxon abundance between groups, the statistical method LinDA (Linear Discriminant Analysis) from the “MicrobiomeStat” package, as described by Zhou et al. [52], was used.

## 5. Conclusions

This study indicates that a GFD can contribute to the stability of the bacterial microbiota in CD. Despite the exclusion of gluten, the functionality of microbiota was comparable to that of healthy controls, with no significant differences in microbial richness or diversity. Furthermore, the reduced prevalence of fast-growing bacteria in the GFD group and the changes observed in antibiotic resistance patterns highlight the potential benefits of this diet in modulating the gut microbiota and possibly reducing the risk of antibiotic resistance. However, this study underscores the complexity of the gut microbiota and the need for further research to better understand its long-term implications.

## Figures and Tables

**Table 1:** Biodiversity index values calculated with the logarithmic difference in the relative abundance of each bacterial taxon.

	Group of Study	
Index	Control	Celiac Disease
log Bacillota—log Bacteroidetes	1.253	1.494
log Bacteroides—log *Prevotella*	–0.094	0.617

## Data Availability

The data presented in this study are available upon request from the corresponding author.

## Supplementary Materials

The following supporting information can be downloaded at https://www.mdpi.com/article/10.3390/antibiotics14080785/s1. Table S1: MIC values for the antibiotics tested under aerobic conditions; Table S2: MIC values for the antibiotics tested under anaerobic conditions; Table S3: Top 25 bacterial genera showing the most significant differences in their relative abundance between the Celiac group vs. the Control group; Figure S1: Nutrient consumption kinetics on Biolog®Eco plates.; Figure S2: Student’s T-test of the antibiotics in aerobic conditions; Figure S3: Student’s T-test of the antibiotics in anaerobic conditions.
