# Correction: Morcillo Serrano et al. Effect of a Gluten-Free Diet on the Intestinal Microbiota of Women with Celiac Disease. *Antibiotics* 2025, *14*, 785

**DOI:** 10.3390/antibiotics15101036

**Published:** 2026-10-19

**Authors:** M. Mar Morcillo Serrano, Paloma Reche-Sainz, Daniel González-Reguero, Marina Robas-Mora, Rocío de la Iglesia, Natalia Úbeda, Elena Alonso-Aperte, Javier Arranz-Herrero, Pedro A. Jiménez-Gómez

**Affiliations:** 1 Department of Pharmaceutical and Health Sciences, Universidad San Pablo-CEU, CEU Universities, Urbanización Montepríncipe, 28660 Boadilla del Monte, Spain; mm.morcillo@usp.ceu.es (M.M.M.S.); paloma.rechesainz@ceu.es (P.R.-S.); marina.robasmora@ceu.es (M.R.-M.); pedro.jimenezgomez@ceu.es (P.A.J.-G.); 2 Research Group “Food and Nutrition in Health Promotion (CEU-NutriFOOD)”, Departamento de Ciencias Farmacéuticas y de la Salud, Facultad de Farmacia, Universidad San Pablo-CEU, CEU Universities, Urbanización Montepríncipe, 28660 Boadilla del Monte, Spain; nubeda@ceu.es (N.Ú.); eaperte@ceu.es (E.A.-A.); 3 Departamento de Ciencias Médicas Básicas, Instituto de Medicina Molecular Aplicada (IMMA) Nemesio Díez, Medicine Faculty, Universidad San Pablo-CEU, CEU Universities, Urbanización Montepríncipe, 28660 Boadilla del Monte, Spain; j.arranz3@usp.ceu.es

In the published article [1], there was an error regarding the affiliations of all authors. The correct affiliations are provided below.

^1^  Department of Pharmaceutical and Health Sciences, Universidad San Pablo-CEU, CEU Universities, Urbanización Montepríncipe, 28660 Boadilla del Monte, Spain; mm.morcillo@usp.ceu.es (M.M.M.S.); paloma.rechesainz@ceu.es (P.R.-S.); marina.robasmora@ceu.es (M.R.-M.); pedro.jimenezgomez@ceu.es (P.A.J.-G.)
^2^  Research Group “Food and Nutrition in Health Promotion (CEU-NutriFOOD)”, Departamento de Ciencias Farmacéuticas y de la Salud, Facultad de Farmacia, Universidad San Pablo-CEU, CEU Universities, Urbanización Montepríncipe, 28660 Boadilla del Monte, Spain; nubeda@ceu.es (N.Ú.); eaperte@ceu.es (E.A.-A.)
^3^  Departamento de Ciencias Médicas Básicas, Instituto de Medicina Molecular Aplicada (IMMA) Nemesio Díez, Medicine Faculty, Universidad San Pablo-CEU, CEU Universities, Urbanización Montepríncipe, 28660 Boadilla del Monte, Spain; j.arranz3@usp.ceu.es

* Correspondence: daniel.gonzalezreguero@ceu.es (D.G.-R.); rocio.delaiglesia@ceu.es (R.d.l.I.)

The authors state that the scientific conclusions are unaffected. This correction was approved by the Academic Editor. The original publication has also been updated.
