# Supplementary material for: Effect of a Gluten-Free Diet on the Intestinal Microbiota of Women with Celiac Disease
Source: Antibiotics (Basel). 2025 Aug 2;14(8):785. doi: 10.3390/antibiotics14080785 (PMC12382989; doi:10.3390/antibiotics14080785)
Supplement: Supplementary file 1 [file antibiotics-14-00785-s001.zip › antibiotics-3680446-supplementary.pdf]

Supplementary Material

Table S1. MIC values for the antibiotics tested under aerobic conditions

Group	Sample	AML	AK	CAZ	CN	TS	CR
Control	**V9**	32	16	256	3	0.64	256
Control	**V18**	256	256	256	1	0.64	256
Control	**V21**	256	4	256	1	32	256
Celiac Disease	**V27**	216	0.50	1	0.016	0.125	1.5
Celiac Disease	**V29**	256	6	256	0.016	0.64	12
Control	**V31**	216	0.50	1	0.016	0.125	1.5
Celiac Disease	**V39**	0.016	0.016	0.016	0.016	0.002	0.016
Control	**V42**	6	1.25	0.016	156	0.002	0.016
Celiac Disease	**V50**	0.016	0.016	0.016	0.016	0.002	2
Celiac Disease	**V64**	32	1.5	0.50	0.016	0.32	0.25
Celiac Disease	**V12**	256	12	16	3	32	256
Control	**V15**	0.12	6	16	0.38	0.016	0.16
Control	**V16**	0.016	0.023	0.094	0.25	0.094	0.50
Control	**V58**	3	0.016	0.016	0.016	0.016	0.064
Control	**V53**	256	0.016	0.5	0.38	0.38	1.5
Celiac Disease	**V60**	256	0.016	0.016	0.094	0.032	0.016
Celiac Disease	**V61**	256	0.75	256	3	16	2
Control	**V69**	0.016	4	6	0.75	32	4
Celiac Disease	**V73**	256	1.5	0.016	0.125	0.064	0.38
Control	**V78**	12	2	12	6	32	0.38
Celiac Disease	**V82**	1.5	0.016	0.38	1.5	32	0.016

Table S2. MIC values for the antibiotics tested under anaerobic conditions

Group	Sample	AML	CLV	LEV	FOX	MTZ	CD	CIP	AZM	RD	IMI+ EDTA	IMI
Control	**V9**	8	4	2	32	32	8	2	32	4	0.094	0.380
Control	**V18**	8	4	2	32	32	8	1	16	1	0.094	0.125
Control	**V21**	8	4	8	32	32	8	1	32	1	0.190	0.380
Celiac Disease	**V27**	32	16	8	32	32	8	2	16	4	0.032	0.125
Celiac Disease	**V29**	16	8	8	32	32	8	4	32	2	0.032	0.250
Control	**V31**	8	4	2	32	32	2	1	32	1	0.032	0.125

Celiac Disease	**V39**	8	4	2	32	8	2	2	16	1	0.032	0.125
Control	**V42**	32	16	2	32	32	8	1	32	4	0.380	0.500
Celiac Disease	**V50**	8	4	2	32	32	4	1	16	2	0.032	0.125
Celiac Disease	**V64**	16	8	8	32	32	8	1	32	2	0.094	0.250
Celiac Disease	**V12**	8	4	2	32	32	8	1	16	1	0.190	0.250
Control	**V15**	8	4	4	32	32	8	4	16	1	0.190	0.125
Control	**V16**	32	16	2	32	32	8	2	16	4	0.032	0.380
Control	**V58**	32	16	8	16	32	8	2	16	1	0.032	0.125
Control	**V53**	8	4	2	32	32	8	2	32	4	0.250	0.190
Celiac Disease	**V60**	8	4	2	32	32	8	1	32	1	0.032	0.125
Celiac Disease	**V61**	16	8	2	32	32	8	2	32	4	2	8
Control	**V69**	8	4	2	32	32	8	1	16	1	0.125	0.125
Celiac Disease	**V73**	8	4	8	32	32	8	4	16	4	0.032	0.125
Control	**V78**	8	4	8	32	32	8	4	32	4	0.032	0.125
Celiac Disease	**V82**	16	8	8	32	32	8	4	32	2	0.032	0.125

Table S3. Top 25 bacterial genera showing the most significant differences in their relative abundance between the Celiac group vs. the Control group. Differences were determined using the LinDA (Linear Discriminant Analysis) method.

Celiac Disease vs Control Variable	p-value	Prevalence	Coefficient	Control Mean Abundance	Celiac Mean Abundance
Oxalobacter	0.0064***	0.4762	3.9346	0.000049	0.000311
Acetanaerobacterium	0.0077***	0.2857	-1.3768	0.000035	0.000000
Paraprevotella	0.0201**	0.4286	4.4880	0.000210	0.000914
Roseburia	0.0348**	0.9524	1.5513	0.014526	0.032766
Noviherbaspirillum	0.0367**	0.1905	1.0198	0.000000	0.000046
Muricomes	0.0367**	0.4286	1.6301	0.000006	0.000029
Caldicoprobacter	0.0427**	0.8571	2.6700	0.000673	0.000832

Sarcina	0.0451**	0.1905	1.9900	0.000000	0.000138
Enterocloster	0.0455**	0.9524	1.3426	0.001903	0.004285
Dysgonomonas	0.0457**	0.1905	0.8897	0.000000	0.000043
Konateibacter	0.0501*	0.9048	1.4248	0.000382	0.001045
Emergencia	0.0502*	0.7619	2.2252	0.000188	0.000597
Terrisporobacter	0.0527*	0.8571	2.8087	0.001724	0.003743
Lacrimispora	0.0541*	0.9524	1.1212	0.002122	0.005158
Marseillibacter	0.0545*	0.9524	1.9763	0.009872	0.013459
Solibaculum	0.0549*	0.8571	2.3275	0.000874	0.001420
Streptococcus	0.0558*	0.9524	1.8795	0.006839	0.018516
Brassicibacter	0.0666*	0.4286	1.2887	0.000008	0.000024
Veillonella	0.0727*	0.7619	1.9953	0.000264	0.001234
Subdoligranulum	0.0754*	0.5714	1.6355	0.000035	0.000119
Ruminococcoides	0.0758*	0.9048	2.1450	0.009565	0.008684
Caecibacterium	0.0805*	0.4286	-2.1887	0.000039	0.000008
Catenibacillus	0.0811*	0.7143	-1.5012	0.000318	0.000153
Lutispora	0.0838*	0.7619	1.9253	0.000623	0.000476
Romboutsia	0.0874*	0.9524	2.9667	0.036482	0.052045

Table S4. Inclusion criteria for the study.

Inclusion criteria	
**Control**	• Not diagnosed with any chronic disease<br>• Not having symptoms or signs of digestive disease on a regular basis<br>• Not taking nutritional supplements
**Celiacs**	• Confirmed diagnosis of celiac disease<br>• Adherence to a gluten-free diet for more than one year<br>• Absence of associated diseases<br>• Not taking nutritional supplements

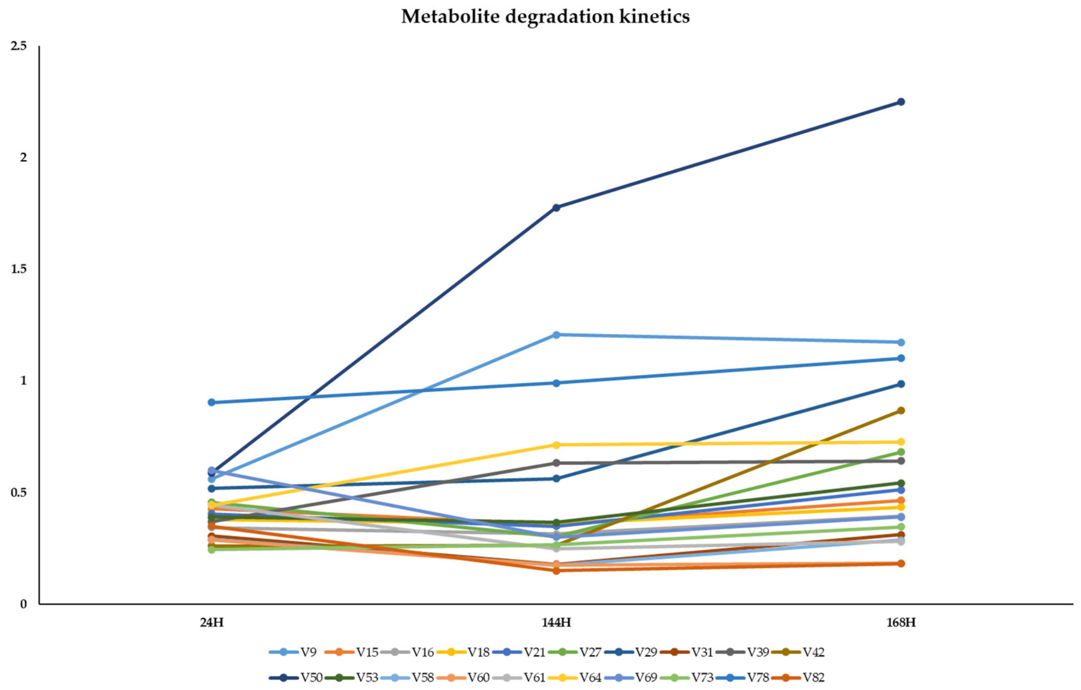

Figure S1. Nutrient consumption kinetics on Biolog®Eco plates. expressed in absorbance units from the AWCD value. Representation of the mean absorbance values measured at 590nm. No complete kinetic curve is shown.

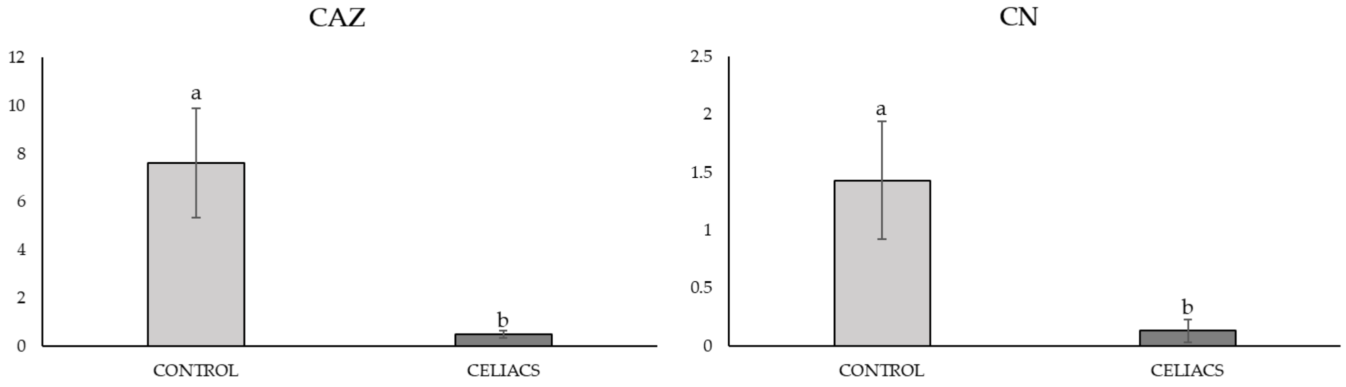

Figure S2. Student's T-test of the antibiotics. CAZ and CN. which shown statistical differences ($p<0.05$) tested in aerobiosis between both study groups.

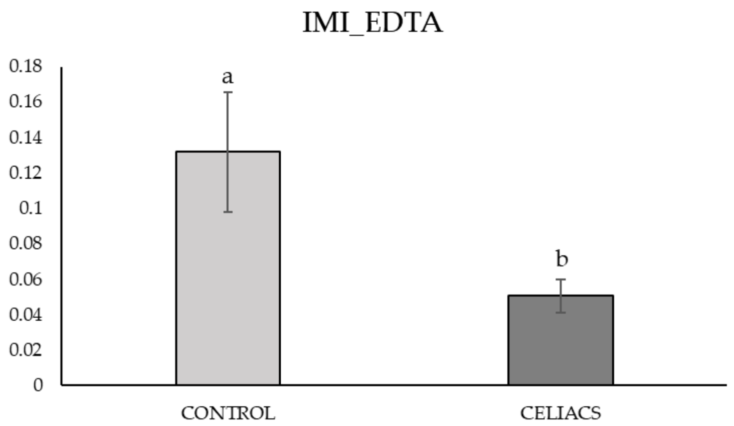

Figure S3. Student's T-test of the antibiotics. IMI_EDTA. which shown statistical differences ($p<0.05$) tested in anaerobiosis between both study groups.
